# The role of lycopene in alleviating soybean meal-induced intestinal injury in an early-weaned piglet model

**DOI:** 10.3389/fvets.2025.1552482

**Published:** 2025-06-13

**Authors:** Shugui Zheng, Mingbo Li, Xinhong Luan, Chuan Tong, Jiguang Li, Zhiying Zhang

**Affiliations:** College of Animal Science and Veterinary Medicine, Shenyang Agricultural University, Shenyang, Liaoning, China

**Keywords:** lycopene, soybean meal, intestinal injury, alleviation, piglets

## Abstract

**Introduction:**

Soybean meal (SBM), widely used in pig farming, can induce intestinal damage and dysfunction in newly-weaned piglets due to the presence of soybean antigen proteins. However, research on natural compounds capable of mitigating these adverse effects remains limited. This study investigated the effects of lycopene, a carotenoid, on the intestinal health of piglets fed a SBM-based diet.

**Methods:**

Eighteen 21-day-old piglets were randomly assigned to three treatment groups: a negative control (NC) group fed an animal protein-based diet, a positive control (PC) group fed a SBM-based diet, and a lycopene group, which received the PC diet supplemented with lycopene. Growth performance, antioxidant capacity, cell apoptosis, intestinal barrier function, intestinal histomorphology, and gut microbiota composition of piglets in each group were systematically evaluated.

**Results:**

Dietary lycopene significantly improved average daily gain (ADG) and average daily feed intake (ADFI) while reducing incidence of diarrhea in early-weaned piglets. Lycopene enhanced antioxidant capacity, as evidenced by increased superoxide dismutase (SOD) activity and total antioxidant capacity (T-AOC) in body. Additionally, lycopene reduced apoptosis in small intestinal cells and strengthened intestinal barrier function, as indicated by decreased serum diamine oxidase (DAO) levels. It also improved small intestinal histomorphology, characterized by increased villus height and reduced crypt depth. Furthermore, lycopene modulated gut microbiota by promoting the growth of beneficial bacteria, particularly Lactobacillus, while reducing the abundance of harmful bacteria.

**Conclusion:**

These findings demonstrated that lycopene supplementation in SBM-based diets significantly enhanced antioxidant capacity, decreased apoptosis in small intestinal cells, improved intestinal barrier function and morphology, and optimized gut microbiota composition. These beneficial effects collectively contributed to improved intestinal health and enhanced production performance in piglets fed a SBM-based diet.

## 1 Introduction

Soybean meal (SBM), a by-product derived from the extraction of soybean oil, is widely used as a high-quality vegetable protein source in the livestock industry, particularly in pig production. Its popularity stems from its high protein content and well-balanced amino acid profile, making it a valuable resource for animal nutrition (1). However, SBM contains several anti-nutritional factors, among which soybean antigen proteins, particularly glycinin and *β*-conglycinin, are the most significant. These proteins can pose serious health risks to piglets, including disruption of intestinal integrity. This disruption manifests as structural damage to the mucosa, villus atrophy, increased crypt depth, and elevated intestinal mucosal permeability, as demonstrated in previous studies (2–4). Additionally, these antigen proteins can trigger allergic reactions, further compromising the overall health of piglets. For instance, glycinin and *β*-conglycinin have been found to elicit immune responses, leading to elevated serum IgG and IgE levels, as well as increased mast cell populations in intestinal mucosal tissue (5–7). Consequently, these adverse effects result in reduced growth efficiency and diarrheal symptoms in early-weaned piglets, significantly impairing their overall health status (8). Therefore, to promote overall health and enhance the efficient utilization of SBM in piglet diets, it is crucial to identify effective natural compounds that can mitigate the negative impacts of SBM on piglets during early weaning.

Lycopene, a carotenoid pigment present in tomatoes and various other fruits, functions as a red colorant (9). Its molecular structure consists of a linear hydrocarbon chain featuring two non-conjugated and eleven conjugated double bonds (10). Lycopene exhibits potent antioxidant properties (11). Furthermore, as a naturally occurring compound, lycopene has been demonstrated to possess a range of biological functions, including the alleviation of cardiovascular diseases (12), aging (13), obesity, and diabetes (14). Although present research has shown that lycopene can enhance intestinal health in piglets experiencing weaning stress (15), studies on the application of lycopene in piglet diets remain insufficient. Specifically, there is a lack of conclusive evidence regarding whether lycopene supplementation in feed can mitigate SBM-induced intestinal injury. Therefore, this study aimed to comprehensively investigate the effects of lycopene on SBM-induced intestinal injury in early-weaned piglets and to elucidate the underlying mechanisms. The goal was to provide experimental data to support the development of novel feed supplements, ultimately improving the gastrointestinal health of piglets during early weaning.

## 2 Materials and methods

### 2.1 Animal experimental protocol

Mixed-breed piglets (Duroc × Landrace × Large White), aged 18 days, were obtained from Shenyang Risun Yijia Animal Husbandry Co., Ltd. for this study. The piglets were housed individually with *ad libitum* access to food and water. They were maintained in a controlled environment with an ambient temperature ranging from 22 to 27°C and a relative humidity of 50–60%. All experimental procedures were conducted in compliance with ethical guidelines (GB/T 35892-2018) and were approved by the Experimental Animal Welfare and Ethics Review Committee of Shenyang Agricultural University.

Three experimental diets were formulated in accordance with the piglet nutrition requirement recommended by NRC (2012) (Table 1). The negative control (NC) diet primarily consisted of casein, skimmed milk powder, and fish meal as the main protein sources; the positive control (PC) diet used soybean meal (SBM) as its main protein source; the lycopene diet was formulated by replacing 0.05% zeolite powder in the PC diet with lycopene (purity >96%), which was obtained from Nanjing Jingzhu Biotechnology Co., Ltd. All feed ingredients were obtained from Shenyang Xinhuakang Feed Co., Ltd.

**Table 1:** Composition and nutritional contents of diets (as fed basis).

Ingredients	Negative control (NC) group (%)	Positive control (PC) group (%)	Lycopene group (%)
Casein	8.00	3.00	3.00
Fish meal	5.00	2.00	2.00
Skimmed milk powder	18.00	6.00	6.00
Soybean meal	-	22.00	22.00
Corn	55.00	55.00	55.00
Whey powder	11.00	3.50	3.50
Soybean oil	-	3.00	3.00
Calcium lactate	1.40	2.10	2.10
Monocalcium phosphate	0.40	1.60	1.60
Feed-grade sodium chloride	0.30	0.30	0.30
L-Lysine-HCl	0.05	0.35	0.35
DL-Methionine	-	0.16	0.16
L-Threonine	-	0.14	0.14
Choline chloride	0.10	0.10	0.10
Vitamin Premix	0.03	0.03	0.03
Mineral Premix	0.12	0.12	0.12
Zeolite powder	0.60	0.60	0.55
lycopene	-	-	0.05
Total	100	100	100
Nutritional levels			
DE (MJ/kg)	14.77	14.79	14.79
ME (MJ/kg)	14.16	14.17	14.17
NE (MJ/kg)	10.68	10.65	10.65
CP (%)	20.65	20.52	20.52
Calcium (%)	0.83	0.82	0.82
STTD phosphorus (%)	0.59	0.59	0.59
SID Lysine (%)	1.37	1.35	1.35
SID Methionine (%)	0.50	0.51	0.51
SID Tryptophan (%)	0.24	0.22	0.22
SID threonine (%)	0.81	0.83	0.83

Vitamin premix supplied per kilogram of complete diet: Vitamin A, 12000 IU; Vitamin D3, 2,400 IU; Vitamin E, 25.50 mg; Vitamin K, 2.88 mg; Vitamin B1, 2.88 mg; Vitamin B2, 7.20 mg; Vitamin B6, 5.84 mg; Vitamin B12, 0.024 mg; Pantothenic acid, 13.50 mg; Niacin/nicotinamide, 31.44 mg; Folic acid, 0.58 mg; Biotin, 0.30 mg. Mineral premix supplied per kilogram of complete diet: copper, 10.80 mg; Iron, 66.00 mg; Zinc, 66.00 mg; Manganese, 16.80 mg; Selenium, 0.36 mg; Iodine, 1.20 mg. The crude protein, calcium, phosphorus, glycinin and β-conglycinin in the diet were measured values, while the others were all calculated values.

After a three-day adaptation period, eighteen 21-day-old crossbred piglets, with an average body weight of 5.18 ± 0.11 kg, were randomly allocated into three treatment groups: the negative control (NC) group, the positive control (PC) group and the lycopene group. Each group consisted of six replicates, with one piglet per replicate. The piglets in the NC group was fed the NC diet, while those in the PC group and the lycopene group were provided with the PC diet and the lycopene diet, respectively. The experiment lasted for 31 days, during which individual piglets were weighed on the first and final days. Daily feed intake was recorded for each piglet to calculate the average daily gain (ADG) and the feed-to-gain ratio (F/G). Throughout the experimental period, the occurrence of diarrhea in piglets was monitored and recorded. Diarrhea was scored based on standardized criteria: 1 for normal, cylindrical feces; 2 for soft feces; 3 for unformed, porridge-like feces; and 4 for watery diarrhea. Scores of 3 or 4 were classified as diarrhea. The incidence of diarrhea was calculated using the following formula: the incidence of diarrhea (%) = (number of diarrhea piglets per group × number of diarrhea days) / (total number of piglets per group × total experimental days) × 100.

### 2.2 Sample collection

Blood samples were collected from the precaval veins of the piglets on day 31. The sera were separated and stored at −20°C for subsequent analysis. All piglets were euthanized on day 31 through intravenous administration of pentobarbital sodium (90 mg/kg) followed by jugular exsanguination, in accordance with the protocols specified in GB/T 39760-2021.

The middle segments of the duodenum, jejunum, and ileum were collected. After rinsing the intestinal contents with pre-cooled physiological saline, the samples were fixed in a 4% paraformaldehyde solution for subsequent examination of intestinal mucosal structure and cellular apoptosis. Tissue samples from the mid-jejunum were rinsed with cooled physiological saline to remove residual digesta, rapidly frozen in liquid nitrogen, and stored at −80°C for later analysis of antioxidant indices. Additionally, digesta samples from the mid-colons were placed in sterile, enzyme-free cryovials, flash-frozen in liquid nitrogen, and stored at −80°C for subsequent microbial sequencing and volatile fatty acid (VFA) quantification.

### 2.3 Measurement of antioxidant indices in sera and small intestinal issues

The concentrations of total antioxidant capacity (T-AOC), superoxide dismutase (SOD), and malondialdehyde (MDA) in the sera and jejunal issues were quantitatively measured using commercial kits according to the manufacturer’s instructions (Elabscience Biotechnology Co., Ltd., Nanjing, China).

### 2.4 Apoptosis detection assay

Apoptotic jejunal cells were quantified using a TUNEL assay kit (Servicebio, Wuhan) according to the manufacturer’s instructions. Briefly, 4-μm-thick paraffin-embedded tissue sections were deparaffinized, rehydrated, and treated with proteinase K at 37°C. The sections were then incubated with the TUNEL reaction mixture for 2 h at 37°C in a humidified chamber. After washing with phosphate-buffered saline (PBS, pH 7.4), the sections were stained with DAPI for 10 min at room temperature in the dark. Finally, coverslips were mounted, and the slides were examined under a fluorescence microscope for image acquisition.

### 2.5 Detection of DAO in sera

The enzymatic activities of diamine oxidase (DAO) in sera were quantitatively determined using a color-based assay method following the manufacturer’s instructions (Elabscience Biotechnology Co., Ltd., Nanjing, China).

### 2.6 Analysis of histomorphological characteristics in small intestinal mucosa

To assess the histomorphological characteristics in small intestinal mucosa, the protocol described by Li et al. (16) was used. In brief, small intestinal segments a were fixed in 4% paraformaldehyde. Following dehydration and embedding, the samples were sectioned into thin slices using a microtome. These sections were then dewaxed and stained with hematoxylin and eosin. Observations and imaging were performed using a Nikon Eclipse E100 microscope (Nikon, Tokyo, Japan). For each slide, thirty intact and well-oriented crypt-villus units were randomly selected, and villus height and crypt depth were measured using the Nikon DS-U3 imaging system (Nikon, Tokyo, Japan).

### 2.7 Analysis of volatile fatty acids (VFAs)

After thawing, colonic digesta samples were homogenized for at least 30 s using a sterile rod. A 1.0-gram aliquot was transferred into a 15-mL centrifuge tube and mixed with 9 mL of deionized water. The mixture was then agitated for 3 to 5 min. Following centrifugation at 5000 × g for 10 min at 4°C, 1 mL of the supernatant was collected and mixed with 0.2 mL of a solution containing 25% metaphosphoric acid and 75 mmol/L crotonic acid. The mixture was vortexed thoroughly and subsequently cooled in an ice-water bath for 30 min. After cooling, the sample was centrifuged at 15000 × g for 10 min and filtered through a 0.22-μm membrane. The filtrate was then analyzed using a gas chromatograph (Agilent, China).

### 2.8 Bacterial DNA extraction, amplification, and sequencing

Total microbial DNA was extracted from colonic digesta using the E.Z.N.A. Soil DNA Kit (Omega Bio-tek, Inc., USA) following the manufacturer’s protocol. The extracted DNA was quantified using a NanoDrop spectrophotometer. PCR amplification was performed targeting the V3-V4 regions of the 16S rRNA gene with the following primers: forward primer 5’-ACTCCTACGGGAGGCAGCAG-3′ and reverse primer 5’-GGACTACHVGGGTWTCTAAT-3′. The amplified DNA products were purified using the AxyPrep DNA Gel Extraction Kit, pooled at equimolar concentrations, and sequenced on the Illumina MiSeq/Novaseq 6000 platform according to the manufacturer’s instructions. Data processing was conducted using the QIIME2 software package. Sequences were clustered into operational taxonomic units (OTUs) at a 97% similarity threshold, and microbial taxa were identified by comparing the sequences with the NR or NT databases. The relative abundance of intestinal bacteria at different taxonomic levels was evaluated using the Kruskal-Wallis test, followed by Bonferroni’s post-hoc test for multiple comparisons.

### 2.9 Statistical analysis

Statistical analysis was performed using one-way analysis of variance (ANOVA) followed by Duncan’s test (SPSS 25). The incidence of diarrhea was analyzed using the chi-square test. Data are presented as mean ± standard deviation (SD), with *p* < 0.05 considered statistically significant.

## 3 Results

### 3.1 Performance and incidence of diarrhea

Table 2 indicated significant differences in average daily feed intake (ADFI) among the three groups of piglets (*p* < 0.05). Specifically, the ADFI of the NC group was significantly higher than that of the lycopene group (*p* < 0.05), while the lycopene group exhibited a significantly higher ADFI compared to the PC group (*p* < 0.01). Piglets in the lycopene group showed a significantly higher average daily gain (ADG) compared to those in the PC group (*p* < 0.05), but their ADG was similar to that of the NC group (*p* > 0.05). No significant differences were observed in the feed-to-gain (F/G) ratios among the three groups (*p* > 0.05). Notably, the incidence of diarrhea was significantly lower in the lycopene group compared to the PC group (*p* < 0.05).

**Table 2:** Impact of lycopene on performance and diarrhea in piglets fed SBM diet.

Items	Negative control (NC) group	Positive control (PC) group	Lycopene group	*p* values
ADG (g)	409.03 ± 25.94^a^	324.95 ± 9.15^b^	382.34 ± 4.91^a^	0.031
ADFI (g)	686.10 ± 6.28^a^	577.89 ± 4.79^c^	612.33 ± 7.62^b^	<0.001
F/G (g/g)	1.70 ± 0.13	1.78 ± 0.06	1.60 ± 0.03	0.406
Incidence of diarrhea (%)	0	16.13	7.10	<0.05

ADFI, average daily feed intake; ADG, average daily gain; F/G, feed-to-gain ratio. Values are shown as mean ± standard deviation (SD), *n* = 6. The diverse letters in the superscripts within the same line denote significant differences (*p* < 0.05). The significance of difference in incidence of diarrhea was analyzed using chi-square test.

### 3.2 Antioxidant indices in sera and small intestinal issues

As shown in Table 3, although there were no significant differences in serum total antioxidant capacity (T-AOC) among piglets in the three groups, the lycopene group exhibited a significant increase in T-AOC levels in jejunal tissue compared to the PC group (*p* < 0.05), but a decrease compared to the NC group (*p* < 0.05). Additionally, the piglets in the lycopene group exhibited significantly higher superoxide dismutase (SOD) levels in both serum and jejunal tissues compared to the PC group (*p* < 0.05). However, there were no significant differences in malondialdehyde (MDA) content in either serum or jejunal tissues among piglets in all three groups (*p* > 0.05).

**Table 3:** Impact of lycopene on antioxidant indices in piglets fed SBM diet.

Samples	Item	Negative control (NC) group	Positive control (PC) group	Lycopene group	*p* value
Jejunal tissue	T-AOC (U/mgprot)	1.09 ± 0.15^a^	0.45 ± 0.13^c^	0.61 ± 0.03^b^	0.019
	SOD (U/mgprot)	126.28 ± 7.75^a^	89.15 ± 2.09^b^	108.49 ± 5.26^a^	0.009
	MDA (μmol/gprot)	0.69 ± 0.01	0.43 ± 0.20	0.39 ± 0.11	0.334
Serum	T-AOC (U/mL)	4.77 ± 1.30	2.01 ± 0.21	2.55 ± 0.58	0.118
	SOD (U/mL)	56.92 ± 3.26^b^	51.94 ± 1.77^b^	66.10 ± 1.96^a^	0.016
	MDA (μmol/L)	1.66 ± 0.29	2.21 ± 0.11	2.32 ± 0.11	0.101

T-AOC, total antioxidant capacity; SOD, superoxide dismutase; MDA, malondialdehyde. Values are shown as mean ± standard deviation (SD), *n* = 6. The diverse letters in the superscripts within the same line denote significant differences (*p* < 0.05).

### 3.3 Apoptosis in small intestinal cells

**Figure 1:**
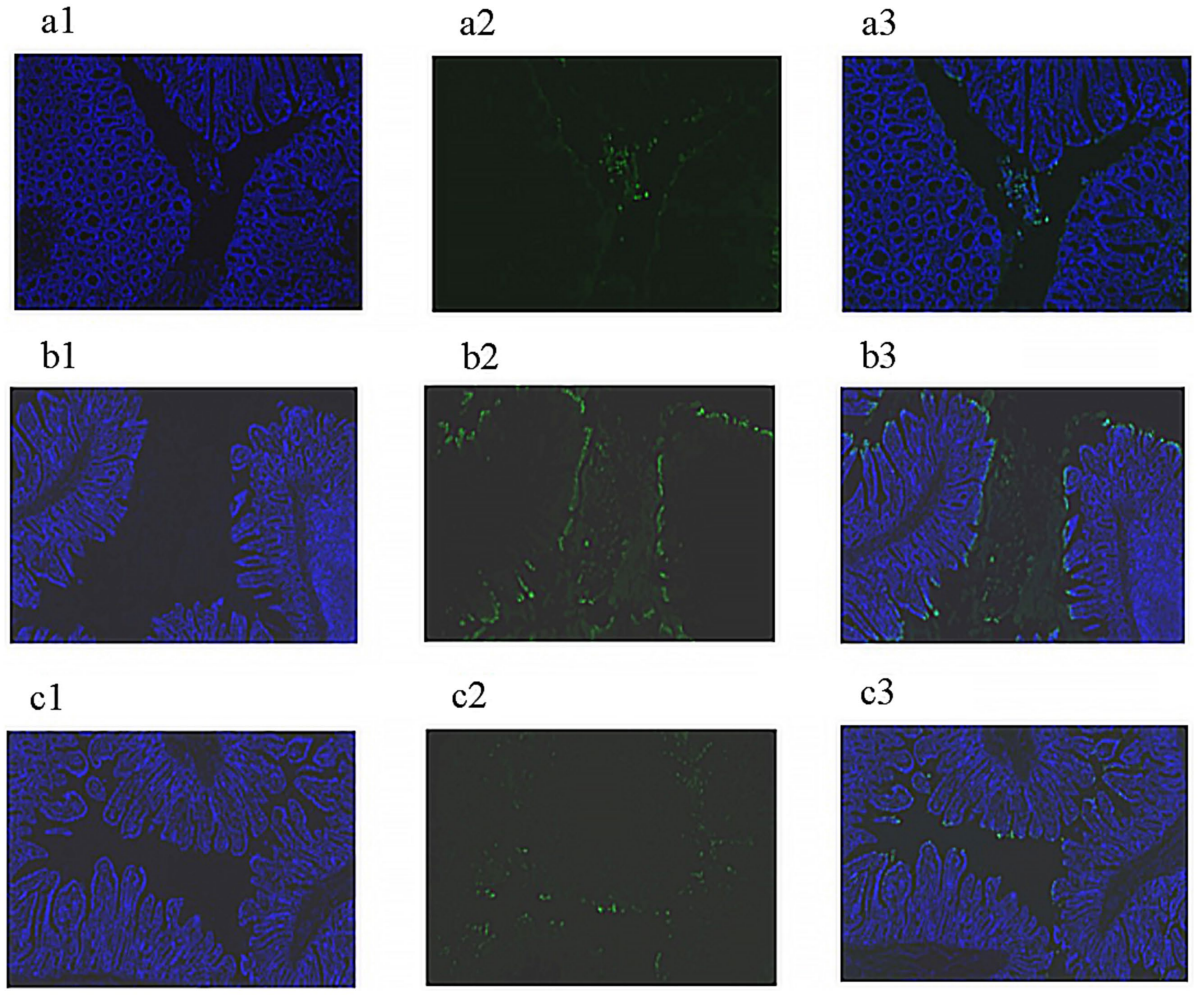
Detection of apoptosis in jejunal cells of early-weaned piglets. **(a)** Negative control (NC) group: **(a1)** DAPI-stained nuclei, **(a2)** FITC-stained apoptotic cells, **(a3)** merged image. **(b)** Positive control (PC) group: **(b1)** DAPI-stained nuclei, **(b2)** FITC-stained apoptotic cells, **(b3)** merged image. **(c)** Lycopene group: **(c1)** DAPI-stained nuclei, **(c2)** FITC-stained apoptotic cells, **(c3)** merged image.

As illustrated in Figure 1, jejunal cells of piglets in the PC group exhibited a significantly higher presence of green fluorescent markers, indicating an elevated level of apoptosis. In contrast, jejunal cells of piglets in both the lycopene group and the NC group showed minimal or no green fluorescent markers, suggesting an absence or low level of apoptosis.

### 3.4 Serum diamine oxidase (DAO) activity

**Figure 2:**
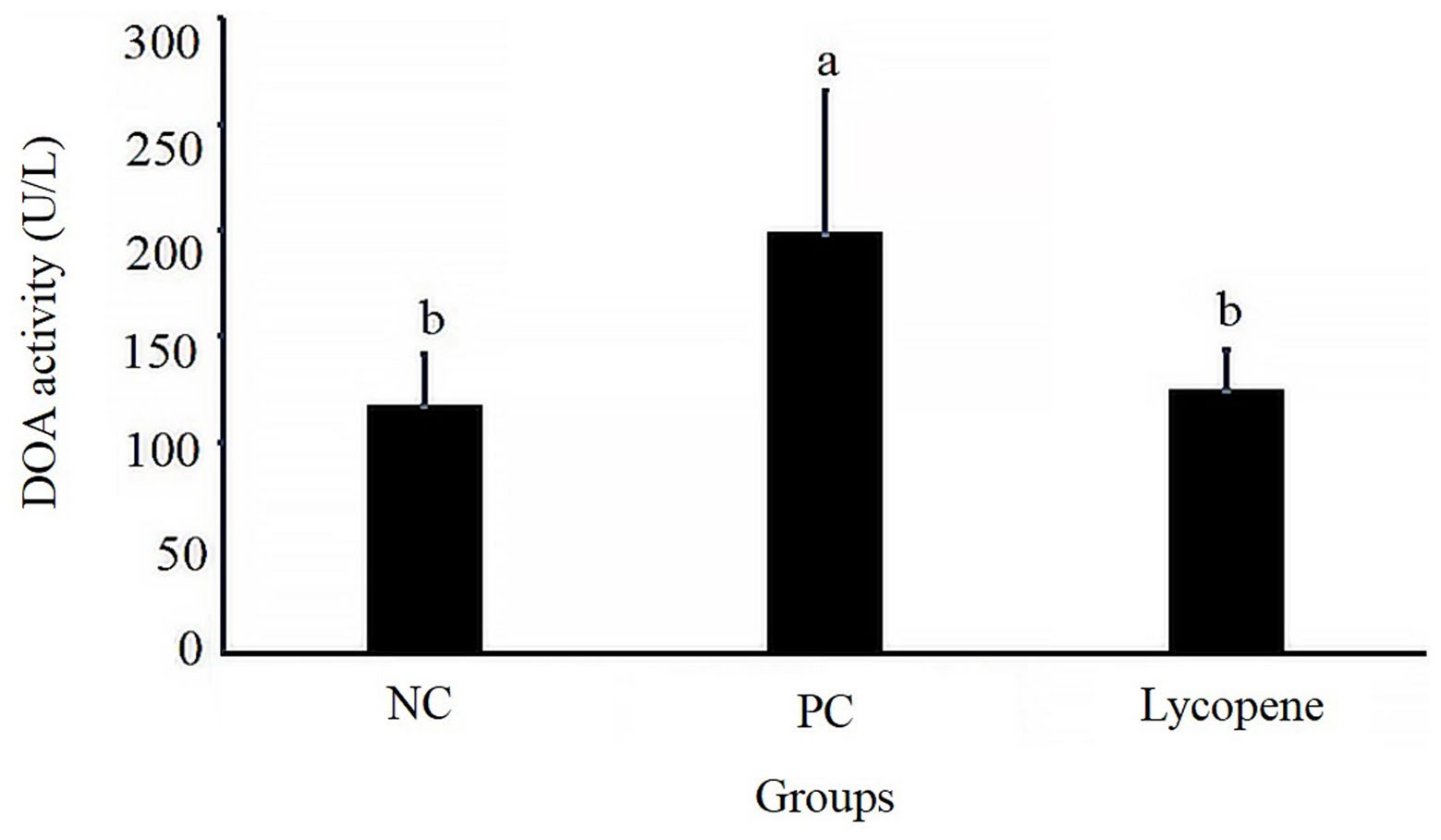
Effect of lycopene on serum diamine oxidase (DAO) activity in early-weaned piglets. Significant differences (*p* < 0.05) are denoted by different superscript lowercase letters among groups, *n* = 6.

As shown in Figure 2, the serum DAO activity in piglets from the lycopene group was significantly lower compared to the PC group (*p* < 0.05), but no significant difference was observed relative to the NC group (*p* > 0.05). These results indicate that lycopene supplementation significantly reduced serum DAO activity in piglets fed a SBM-based diet.

### 3.5 Histomorphology of small intestinal mucosa

**Figure 3:**
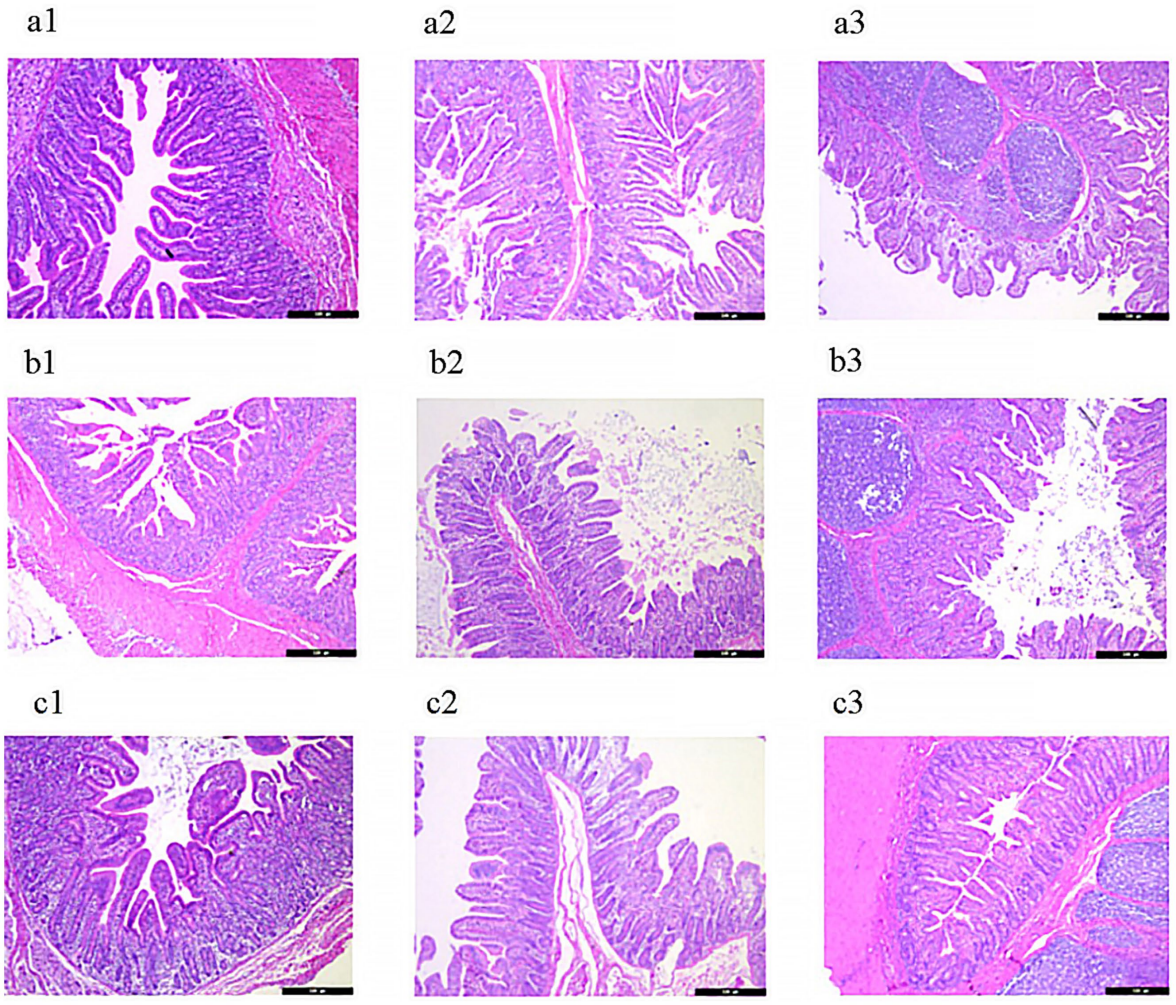
Representative HE-stained images of intestinal mucosa in early-weaned piglets. **(a)** Negative control (NC) group: **(a1)** duodenum, **(a2)** jejunum, **(a3)** ileum. **(b)** Positive control (PC) group: **(b1)** duodenum, **(b2)** jejunum, **(b3)** ileum. **(c)** Lycopene group: **(c1)** duodenum, **(c2)** jejunum, **(c3)** ileum.

As shown in Figure 3 and Table 4, piglets in the lycopene group exhibited a significant improvement in duodenal villus height and the villus-to-crypt ratio compared to those in the PC group (*p* < 0.05), although these parameters were similar to those in the NC group (*p* > 0.05). Among the three treatment groups, no significant differences were observed in jejunal villus height, crypt depth, or the villus-to-crypt ratio (*p* > 0.05). In the ileum, while villus height did not differ significantly among piglets from the three groups (*p* > 0.05), the lycopene group showed a significantly reduced crypt depth compared to the PC group (*p* < 0.05), but the crypt depth was comparable to that of the NC group (*p* > 0.05).

**Table 4:** Impact of lycopene on small intestinal mucosal histomorphology in piglets fed SBM diet.

Samples	Item	Negative control (NC) group	Positive control (PC) group	Lycopene group	*p* value
Duodenum	Crypt depth (μm)	359.53 ± 28.38	426.24 ± 29.76	379.11 ± 18.68	0.255
	Villus height (μm)	559.69 ± 69.11^a^	416.05 ± 30.88^b^	615.56 ± 18.61^a^	0.048
	Villus to crypt ratio	1.58 ± 0.10^a^	1.00 ± 0.02^b^	1.74 ± 0.12^a^	0.003
Jejunum	Crypt depth (μm)	245.53 ± 14.90	290.19 ± 15.73	289.69 ± 25.60	0.251
	Villus height (μm)	394.72 ± 69.59	332.32 ± 43.77	301.17 ± 45.42	0.503
	Villus to crypt ratio	1.67 ± 0.36	1.20 ± 0.21	1.08 ± 0.18	0.301
Ileum	Crypt depth (μm)	182.77 ± 8.92^b^	257.94 ± 15.90^a^	209.10 ± 14.05^b^	0.019
	Villus height (μm)	384.61 ± 27.90	269.68 ± 21.70	311.57 ± 48.34	0.136
	Villus to crypt ratio	2.20 ± 0.04^a^	1.08 ± 0.04^b^	1.66 ± 0.39^ab^	0.037

Values are shown as mean ± standard deviation (SD), *n* = 6. The diverse letters in the superscripts within the same line denote significant differences (*p* < 0.05).

### 3.6 Intestinal volatile fatty acids (VFA)

As presented in Table 5, no significant differences were observed in the levels of acetic acid, propionic acid, or butyric acid in the colonic digesta of piglets among the lycopene group, PC group, and NC group (*p* > 0.05). These results indicated that lycopene supplementation had no significant effect on intestinal VFA levels in piglets.

**Table 5:** Impact of lycopene on volatile fatty acids (VFA) in colon contents of piglets fed SBM diet (mmol/L).

Items	Negative control (NC) group	Positive control (PC) group	Lycopene group	*P* value
Butyric acid	13.56 ± 0.93	15.87 ± 0.73	13.95 ± 4.48	0.299
Propanoic Acid	30.23 ± 0.56	29.86 ± 1.60	26.92 ± 0.83	0.171
Acetic Acid	105.56 ± 10.06	101.15 ± 7.06	104.52 ± 2.90	0.915

Values are denoted as mean ± standard deviation (SD), *n* = 6.

### 3.7 Colonic microbiota composition

**Figure 4:**
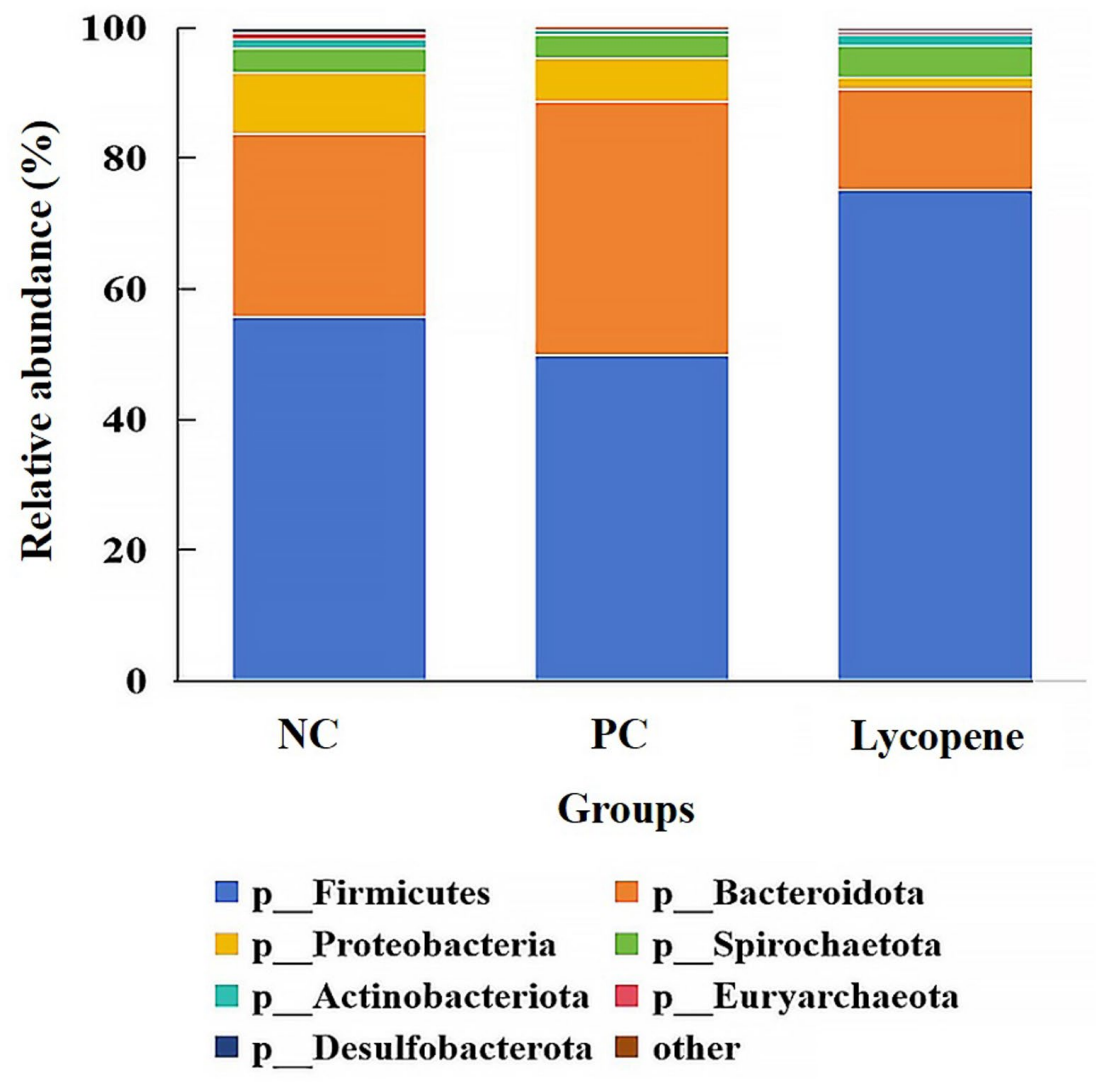
Relative abundance of colonic bacteria at the phylum level in early-weaned piglets. “Others” represents the combined abundance of the remaining phyla.

To further investigate the effects of lycopene on the intestinal microbiota of piglets, 16S rDNA sequencing was performed on colonic digesta. As shown in Figure 4, the predominant bacterial phyla identified in the NC group, PC group, and lycopene group were Firmicutes, Bacteroidota, Proteobacteria, Spirochaetota, and Actinobacteriota. These five phyla collectively accounted for over 98% of the total bacterial phyla in all groups. Specifically, the relative abundance of Firmicutes was 55.69, 49.81, and 75.07% in the NC, PC, and lycopene groups, respectively. The abundance of Bacteroidota was 28.07, 38.92, and 15.43%, while Proteobacteria comprised 9.43, 6.60, and 1.79% in the respective groups. Spirochaetota accounted for 3.59, 3.43, and 4.96%, and Actinobacteriota represented 1.37, 0.81, and 1.59%, respectively, as detailed in Table 6. Notably, the relative abundance of Firmicutes in the lycopene group showed an increasing trend compared to the PC group (*p* = 0.055), whereas the relative abundance of Bacteroidota exhibited a decreasing trend relative to the PC group (*p* = 0.059).

**Table 6:** Relative abundances of the dominant bacteria of piglets at phylum level (%).

Phyla	Negative control (NC) group	Positive control (PC) group	Lycopene group	*P* value
Firmicutes	55.69 ± 7.06	49.81 ± 1.85	75.07 ± 6.92	0.055
Bacteroidota	28.07 ± 6.44	38.92 ± 5.25	15.43 ± 5.62	0.059
Proteobacteria	9.43 ± 3.69	6.60 ± 2.55	1.79 ± 1.08	0.116
Spirochaetota	3.59 ± 1.22	3.43 ± 1.36	4.96 ± 4.59	0.584
Actinobacteriota	1.37 ± 0.51	0.81 ± 0.30	1.59 ± 0.74	0.694

Values are denoted as mean ± standard deviation (SD), *n* = 6.

**Figure 5:**
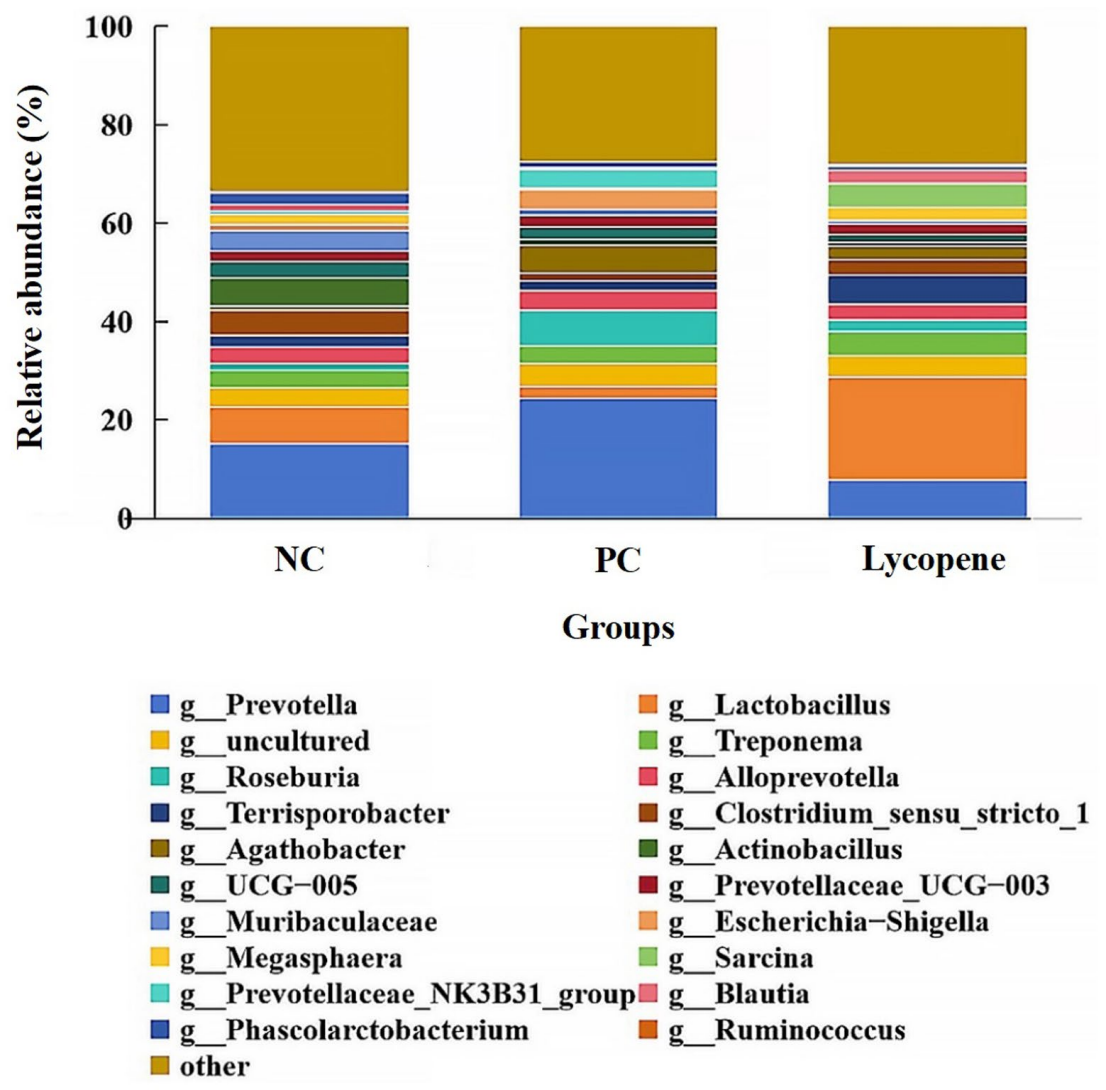
Relative abundance of colonic bacteria at the genus level in early-weaned piglets. “Others” refer to the combined abundance of other genera.

As illustrated in Figure 5, the dominant genera identified in the three groups included *Prevotella*, *Lactobacillus*, *Treponema*, *Roseburia*, *Alloprevotella*, *Actinobacillus*, and *Escherichia-Shigella*, among others. The abundance of *Prevotella* in piglet colons was 15.14% in the NC group, 24.42% in the PC group, and 7.74% in the lycopene group. Similarly, *Lactobacillus* abundances were 7.35, 2.23, and 21.06%, while *Treponema* abundances were 3.59, 3.43, and 4.96%. For *Roseburia*, the abundances were 1.44, 7.42, and 2.41%, and *Alloprevotella* abundances were 3.30, 3.99, and 3.15%. Additionally, *Actinobacillus* abundances were 5.77, 1.24, and 0.76%, and *Escherichia-Shigella* abundances were 1.00, 4.07, and 0.28%, respectively (Table 7). Notably, the abundance of *Lactobacillus* was significantly higher in the lycopene group compared to the PC group (*p* < 0.05). Conversely, the lycopene group exhibited a decreasing trend in the abundance of *Prevotella* relative to the PC group (*p* = 0.063). Similarly, the abundance of *Megasphaera* tended to increase in the lycopene group compared to the PC group (*p* = 0.098). However, the lycopene group showed a tendency for reduced *Escherichia-Shigella* abundance relative to the PC group, although this difference was not statistically significant (*p* = 0.069).

**Table 7:** Relative abundances of the dominant bacteria of piglets at genus level (%).

Genera	Negative control (NC) group	Positive control (PC) group	Lycopene group	*p* value
*Lactobacillus*	7.35 ± 3.19^ab^	2.23 ± 0.66^b^	21.06 ± 7.70^a^	0.044
*Prevotella*	15.14 ± 4.49	24.42 ± 4.10	7.74 ± 4.76	0.063
*Treponema*	3.59 ± 1.22	3.43 ± 1.36	4.96 ± 4.59	0.591
*Roseburia*	1.44 ± 0.39	7.42 ± 3.93	2.41 ± 1.27	0.116
*Alloprevotella*	3.30 ± 1.19	3.99 ± 1.29	3.15 ± 2.54	0.551
*Actinobacillus*	5.77 ± 3.96	1.24 ± 0.88	0.76 ± 0.60	0.491
*Escherichia − Shigella*	1.00 ± 0.24	4.07 ± 1.58	0.28 ± 0.21	0.069
*Megasphaera*	2.34 ± 1.01	0.20 ± 0.14	2.49 ± 2.20	0.098
*Phascolarctobacteriu*	2.37 ± 0.77	1.08 ± 0.53	0.97 ± 0.51	0.232
*Blautia*	1.36 ± 0.07	0.51 ± 0.13	2.57 ± 1.69	0.155
*Ruminococcus*	0.25 ± 0.08	0.11 ± 0.03	0.33 ± 0.12	0.794
*Agathobacter*	0.74 ± 0.33^b^	5.80 ± 2.18^a^	2.72 ± 0.99^ab^	0.026

Values are denoted as mean ± standard deviation (SD), *n* = 6. Data with diverse letters in the superscripts within the same line are significantly different (*p* < 0.05).

## 4 Discussion

Soybean meal (SBM), which is rich in protein and contains a balanced profile of amino acids, is a critical component in animal feed. However, this valuable ingredient also contains anti-nutritional factors, notably soybean antigen proteins such as glycinin and *β*-conglycinin, which present significant challenges in animal nutrition. When consumed by weaned piglets, these antigen proteins can elicit adverse physiological responses. For example, they may induce intestinal damage and allergic reactions, leading to diarrhea, and ultimately resulting in reduced growth rates and impaired overall performance (17–20).

Lycopene, a naturally occurring carotenoid predominantly found in tomatoes, has been scientifically demonstrated to offer significant health benefits. Its unique properties enable it to counteract metabolic disorders and diseases driven by oxidative stress (21). However, comprehensive research is essential to fully harness the advantages of this substance. In this study, we used early-weaned piglets as a model to investigate lycopene’s potential in mitigating intestinal injury induced by SBM. The results showed that lycopene supplementation increased both ADG and ADFI in piglets fed with SBM. Notably, lycopene supplementation also reduced the incidence of diarrhea. These results indicated that lycopene effectively prevented the adverse effects on growth performance and reduced diarrhea in early-weaned piglets induced by SBM.

The body’s response to oxidative stress is often assessed through biomarkers such as superoxide dismutase (SOD), malondialdehyde (MDA), and total antioxidant capacity (T-AOC). SOD, an antioxidant enzyme, neutralizes harmful free radicals by converting superoxide anions into less reactive forms, thereby protecting cells from damage. Its activity serves as an indicator of the body’s ability to combat oxidative stress. MDA, a byproduct of lipid peroxidation, acts as a marker for the extent of lipid oxidation and cellular damage. Elevated MDA levels indicate a higher degree of oxidative stress within cells and tissues. T-AOC represents the body’s overall antioxidant capacity, encompassing both enzymatic and non-enzymatic antioxidants. By measuring T-AOC, researchers can assess the oxidant-antioxidant balance within the body, providing a comprehensive view of oxidative stress status. These biomarkers offer critical insights into the body’s response to oxidative stress and its potential impact on health. The current study demonstrated that lycopene supplementation significantly enhanced SOD activity in both serum and jejunal tissue, while also increasing T-AOC in jejunal tissue. These results indicated that lycopene boosted antioxidant capacity and reduced oxidative damage in early-weaned piglets fed with SBM. These findings were consistent with previous researches (22–24) and further demonstrated the potent ability of lycopene to alleviate oxidative stress.

Soybean antigen proteins in SBM has been shown to hinder the proliferation of intestinal epithelial cells and induce apoptosis in piglets (18). The results of our current experiment demonstrated that dietary supplementation with lycopene significantly inhibited this apoptosis, thereby alleviating the detrimental effects of these soybean antigen proteins. Diamine oxidase (DAO), an enzymatic protein primarily active in the apical villus region of the mammalian intestinal mucosa, plays a critical role in metabolizing histamine and other polyamines. DAO activity is strongly correlated with nucleic acid and protein synthesis in the epithelial cells of the small intestinal mucosa (25). Serum DAO activity serve as a reliable indicator for assessing intestinal barrier function and the extent of intestinal damage (26). In our study, supplementing lycopene into an SBM-based diet significantly reduced serum DAO activity, indicating that lycopene enhanced intestinal barrier function in piglets fed with SBM. These results were consistent with a previous research (15). Changes in the intestinal mucosal morphology of piglets, such as villus atrophy and crypt deepening, impair their digestive and absorptive functions. These morphological alterations directly affect the piglets’ ability to digest and utilize nutrients efficiently, ultimately leading to reduced performance (27). The current study revealed that, compared to the NC group fed with animal protein feed, SBM significantly reduced villus height and the villus-to-crypt ratio in the duodenum, while increasing crypt depth and lowering the villus-to-crypt ratio in the ileum of early-weaned piglets. Notably, lycopene supplementation alleviated the SBM-induced damage to the small intestinal mucosa. This finding demonstrated lycopene’s efficacy in preserving the morphological and structural integrity of the intestinal mucosa, which was consistent with previous researches (15, 28).

Numerous studies have demonstrated a strong correlation between intestinal microbiota and intestinal health in piglets. Changes in the intestinal microbiota of piglets are often accompanied by corresponding alterations in the structure and function of the intestinal mucosa, which can significantly impact the overall health and growth performance of piglets (29, 30). In the current study, the effects of lycopene supplementation on intestinal microbiota in piglets were investigated using 16S rRNA sequencing technology. At the phylum level, the colonic microbiota of piglets was predominantly composed of Firmicutes, Bacteroidota, Proteobacteria, Spirochaetota, and Actinobacteriota, which was consistent with previous scientific observations (18, 31). Firmicutes and Bacteroidota are widely recognized as the two dominant phyla in the intestine, while Proteobacteria, Spirochaetota, and Actinobacteriota also constitute significant portions of the intestinal microbiota. In this study, lycopene supplementation was associated with an increasing trend in the abundance of Firmicutes and a decreasing trend in the abundance of Bacteroidota, suggesting that lycopene may promote the growth of Firmicutes while suppressing the proliferation of Bacteroidota.

At the genus level, the predominant bacteria in the piglets’ colon included *Prevotella*, *Lactobacillus*, *Treponema*, *Roseburia*, *Alloprevotella*, *Actinobacillus*, and *Escherichia-Shigella*, among others. Notably, the abundance of *Prevotella* in the colon of piglets from the lycopene group exhibited a decreasing trend compared to the PC group. *Prevotella* is a common commensal bacterium found in the intestines of both animals and humans (32, 33). However, emerging evidence suggests that certain strains of *Prevotella* may be associated with inflammatory diseases, such as inflammatory bowel disease, indicating potential pathogenicity (34, 35). Further research is needed to clarify the definitive role and potential underlying mechanisms by which *Prevotella* may contribute to disease pathogenesis. In contrast to the piglets in the PC group, the piglets in the lycopene group exhibited a significant increase in the relative abundance of *Lactobacillus*, with a more than 9-fold rise from 2.23 to 21.06%. *Lactobacillus*, a primary beneficial commensal bacterium in the intestines of humans and animals, improves intestinal health through multiple mechanisms. It produces antimicrobial substances that inhibit the colonization of pathogenic bacteria (36), stimulates mucosal immunity (37, 38), and modulates cytokine production (39, 40), collectively contributing to gut health maintenance. Additionally, the relative abundance of *Escherichia-Shigella*, a harmful bacterium associated with diarrhea (41), showed a decreasing trend in the lycopene group compared to the PC group. This reduction in *Escherichia-Shigella* abundance may explain the lower incidence of diarrhea observed in the lycopene group. Conversely, the relative abundance of *Megasphaera* in the lycopene group displayed an increasing trend compared to the PC group. *Megasphaera* is considered as beneficial to host health due to its ability to produce volatile fatty acids (VFAs), vitamins, and essential amino acids, as well as its role in regulating the host’s immune response (42–44). Lycopene may also benefit the intestinal health of early-weaned piglets by inducing an increase in *Megasphaera*.

In conclusion, feeding early-weaned piglets a diet high in soybean meal readily induced oxidative stress, leading to oxidative damage in the epithelial cells of the small intestinal mucosa and subsequently triggering apoptosis. Excessive apoptosis disrupted the epithelial barrier, increased intestinal permeability, and altered mucosal morphology. This study demonstrated that lycopene effectively alleviated oxidative stress and inhibited intestinal cell apoptosis, thereby enhancing barrier function, improving intestinal morphology, and further regulating gut microbiota to restore intestinal health. As a result, lycopene improved production performance and reduced the incidence of diarrhea. These findings supported the efficacy of lycopene as a therapeutic agent for maintaining intestinal health in early-weaned piglets fed SBM, holding great promise in mitigating intestinal injury and dysfunction.

## Data Availability

All raw sequences were submitted to the NCBI Sequence Read Archive under BioProject: PRJNA1267022.
