# Supplementary material for: The role of lycopene in alleviating soybean meal-induced intestinal injury in an early-weaned piglet model
Source: Front Vet Sci. 2025 Jun 13;12:1552482. doi: 10.3389/fvets.2025.1552482 (PMC12202226; doi:10.3389/fvets.2025.1552482)
Supplement: Supplementary file 1 [file Data_Sheet_1.docx]

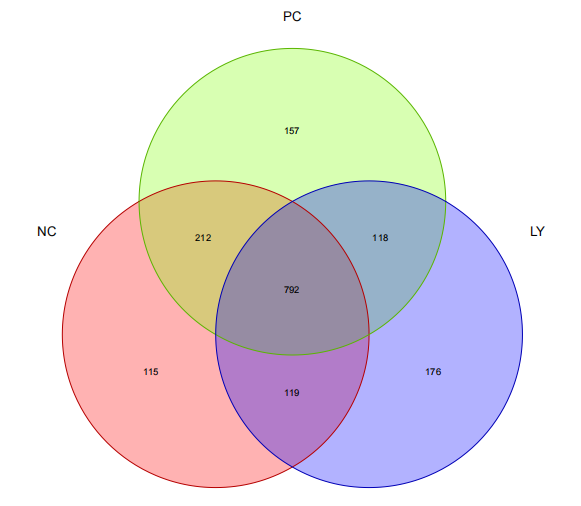


Figure S1. Unique and common colonic OTUs among the negative control, positive control and lycopene group (Venn diagram)

Note: NC, negative control group; PC, positive control group; LY, lycopene group.


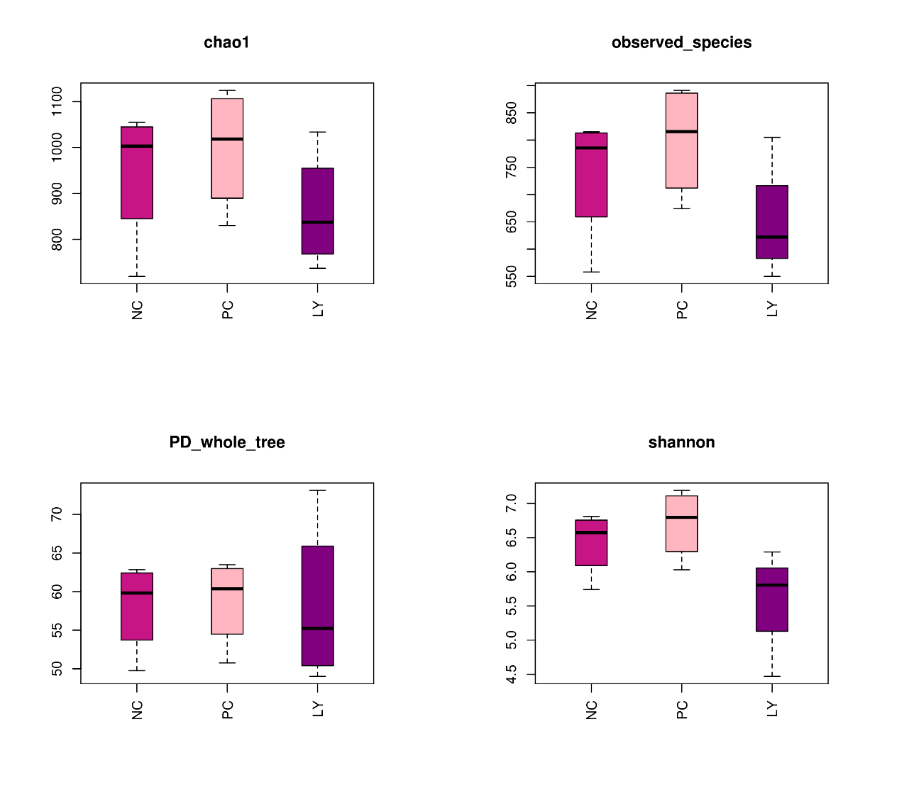


Figure S2. α-diversity of colonic microbial communities in piglets among the negative control, positive control and lycopene group.

Note: NC, negative control group; PC, positive control group; LY, lycopene group.

Table S1. Relative abundances of the dominant bacteria of piglets at species level (%).

| Species | Negative control (NC) group | Positive control (NC) group | Lycopene group | *p* value |
| --- | --- | --- | --- | --- |
| *Lactobacillus*_*prophage* | 3.74±2.05 | 0.46±0.12 | 7.15±6.30 | 0.058 |
| *Actinobacillus*_*porcinus* | 4.53±3.21 | 0.85±0.68 | 0.38±0.33 | 0.309 |
| *Lactobacillus*_*salivarius* | 1.72±0.99 | 0.71±0.26 | 3.26±1.82 | 0.292 |
| *Lactobacillus*_*reuteri* | 1.00±0.24 | 4.07±1.58 | 0.28±0.21 | 0.092 |
| *uncultured*_*Lactobacillus* | 0.41±0.28 | 0.12±0.01 | 0.91±0.15 | 0.232 |
| *Megasphaera*_*elsdenii* | 0.17±0.10 | 0.15±0.06 | 1.07±0.65 | 0.092 |
| *Escherichia_coli* | 2.34±1.01 | 0.20±0.14 | 2.48±2.19 | 0.069 |
